# Resveratrol Ameliorates Mitophagy Disturbance and Improves Cardiac Pathophysiology of Dystrophin-deficient *mdx* Mice

**DOI:** 10.1038/s41598-018-33930-w

**Published:** 2018-10-22

**Authors:** Atsushi Kuno, Ryusuke Hosoda, Rio Sebori, Takashi Hayashi, Hiromi Sakuragi, Mika Tanabe, Yoshiyuki Horio

**Affiliations:** Department of Pharmacology, Sapporo Medical University School of Medicine, Sapporo, Hokkaido, Japan

## Abstract

Autophagy activation improves the phenotype in *mdx* mice, a Duchenne muscular dystrophy (DMD) model, although the underlying mechanisms are obscure. We previously found that resveratrol, a strong inducer of autophagy, ameliorates the cardiac pathology of *mdx* mice. Autophagy could eliminate damaged mitochondria, a major source of intracellular reactive oxygen species (ROS), although there is no evidence for mitochondriopathy in dystrophic cardiomyopathy. To elucidate resveratrol’s function, we investigated the deletion of mitochondrial DNA (mtDNA), autophagy of damaged mitochondria (mitophagy), and ROS accumulation in the *mdx* mouse heart. Low levels of normal mtDNA and abnormal accumulations of mitochondria-containing autophagosomes were found in the *mdx* mouse heart. Administering resveratrol to *mdx* mice for 56 weeks ameliorated the cardiomyopathy, with significant reductions in the amount of mtDNA deletion, the number of mitochondria-containing autophagosomes, and the ROS levels. Resveratrol induced nuclear FoxO3a accumulation and the expression of autophagy-related genes, which are targets of FoxOs. The most effective dose in *mdx* mice was 0.4 g resveratrol/kg food. In conclusion, resveratrol improved cardiomyopathy by promoting mitophagy in the *mdx* mouse heart. We propose that acquired mitochondriopathy worsens the pathology of DMD and is a potential therapeutic target for the cardiomyopathy in DMD patients.

## Introduction

Duchenne muscular dystrophy (DMD), the most common and severe type of muscular dystrophy, is an X-linked lethal disorder^1^. Mutations in the *dystrophin* gene lead to cardiomyopathy and heart failure, which is the main cause of death in DMD patients. Dystrophin deficiency causes intracellular Ca^2+^ dysregulation, resulting in mitochondrial dysfunction and the production of reactive oxygen species (ROS)^2^. Increased ROS levels in turn cause mitochondrial DNA (mtDNA) damage including mtDNA deletion^3^. The mitochondria are a major source of ROS in most cells^4^, and mitochondria with deleted mtDNAs produce high ROS levels^5,6^. Although the deletion of mtDNA in DMD has not been examined, multiple mtDNA deletions are found in human myotonic dystrophy^7^. Persistent cellular damage caused by the vicious cycle of ROS production and mtDNA damage may underlie the development of cardiomyopathy in DMD.

Autophagy is a cell system for discarding damaged proteins and disabled organelles including mitochondria by sequestrating them into a double-membraned autophagosome, which fuses with a lysosome to digest the autophagosomal contents^8^. Autophagy is reported to be impaired in muscular dystrophies, and the activation of autophagy by low-amino-acid intake ameliorates the skeletal muscle phenotype of mouse models of muscular dystrophies including dystrophin-deficient *mdx* mice, a model of DMD^9,10^. However, it remains unknown whether an alteration in mitochondria autophagy (mitophagy) contributes to the pathology of DMD.

SIRT1, an NAD^+^-dependent protein deacetylase^11^, promotes autophagy by deacetylating autophagy-related molecules such as Atg5, Atg7, and Atg8 (LC3)^12^. SIRT1 also deacetylates and activates FoxO transcription factors (FoxOs), which induce the expression of autophagy-related genes and promote autophagy^13–15^. We previously showed that treatment with resveratrol, a SIRT1 activator, significantly reduces the muscle ROS levels^16^ and improves the cardiomyopathy in *mdx* mice^17^. Because resveratrol promotes autophagy in a SIRT1-dependent manner^18^, resveratrol may promote mitophagy in *mdx* mice.

Here, we investigated whether the mitophagy-mediated elimination of damaged mitochondria is impaired in the *mdx* mouse heart. We further examined whether resveratrol administration promotes mitophagy, reduces ROS levels, and ameliorates the cardiac pathophysiology in the *mdx* mice.

## Results

### Mitophagy is disturbed in the *mdx* mouse heart

**Figure 1:**
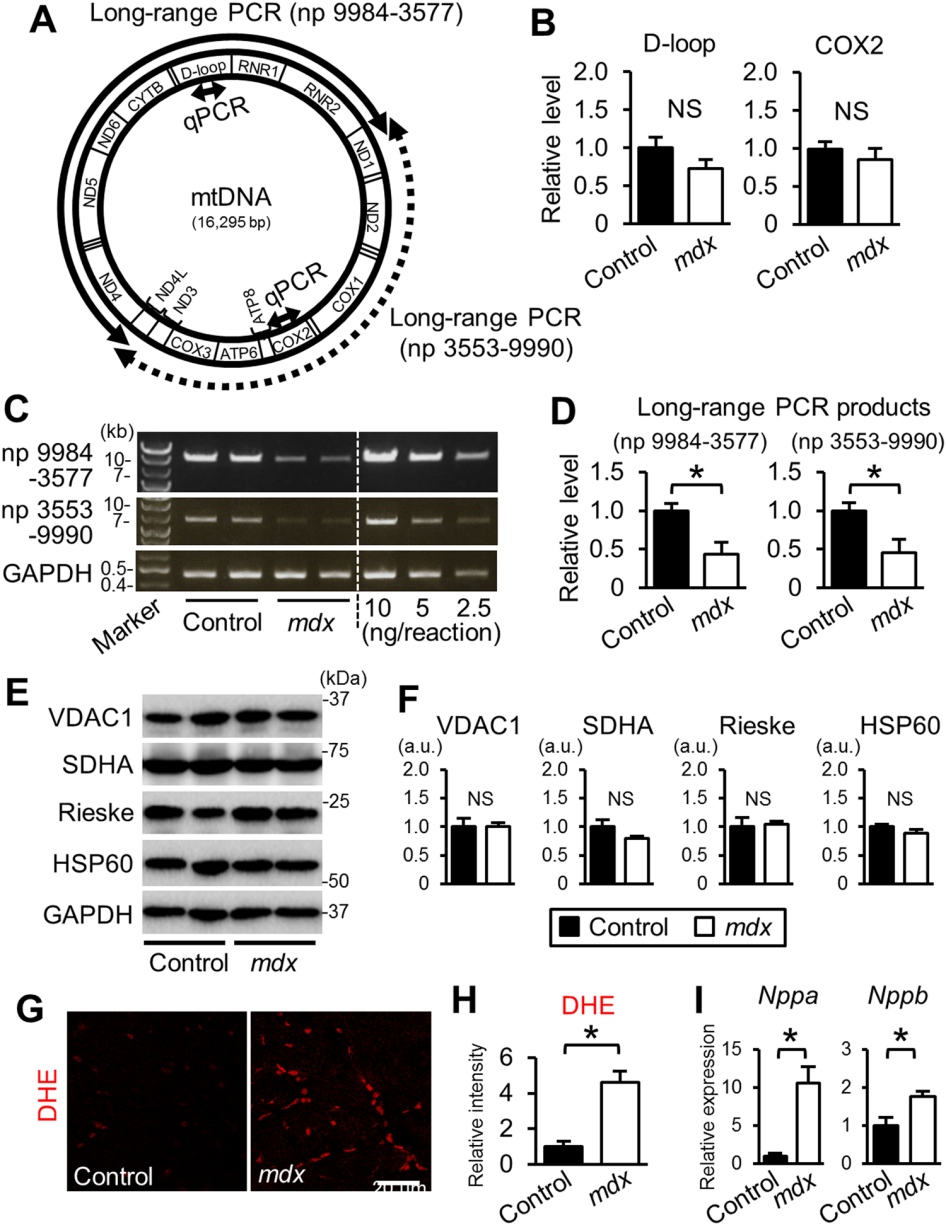
Levels of mtDNA with deletion and tissue ROS were increased in the *mdx* mouse heart. (**A**) Schematic depicting the regions of the mouse mitochondrial genome (mtDNA) amplified by long-range PCR [nucleotide positions (np) 9984-3577 and np 3553–9990] and the qPCR methods. (**B**) mtDNA content determined by qPCR amplifying the D-loop and COX2 regions and nuclear RPS18 genome region. N = 4. (**C**) Representative gel images of long-range PCR of myocardial DNA samples. For quantification, the results of 10, 5, and 2.5 ng of DNA from an intact mouse heart per reaction were included. The nuclear *Gapdh* gene was amplified as an internal control. (**D**) Levels of long-range PCR products normalized to *Gapdh*. N = 4. (**E**) Representative Immunoblots for VDAC1, SDHA, Rieske, HSP60, and GAPDH. (**F**) Levels of mitochondrial proteins in the hearts. (**G**) Dihydroethidium (DHE) fluorescence (red) images in heart sections from 22-week-old control and *mdx* mice. (**H**) Relative DHE fluorescence intensity. Eight images randomly captured from 4 hearts were analyzed in each group. (**I**) qPCR analyses of *Nppa* and *Nppb* genes normalized to β-actin. N = 4. All data were analyzed by unpaired 2-tailed Student’s t test. *P < 0.05. NS: not significant. a.u.: arbitrary units. kb: kilobase.

We first analyzed whether damaged mitochondria are accumulated in the heart of *mdx* mice, a model of DMD. The heart weight to tibia length ratio was similar in dystrophin-deficient *mdx* mice (5.5 ± 0.2 vs. 5.9 ± 0.2 mg/mm, respectively) at 22 weeks old and age-matched control C57BL10 mice, indicating no cardiac hypertrophy in *mdx* mice at this age. We compared the levels of the cardiac mtDNA D-loop and COXII regions of *mdx* mice with those of control mice. Although deletions in the D-loop mtDNA region in hydrogen-peroxide-treated cells are reported^19^, qPCR revealed no differences in the D-loop or COXII mtDNA region between *mdx* and control mice (Fig. 1A,B). Amplification of the mtDNA region from nucleotide position (np) 9984 to np 3577 by long-range PCR (Fig. 1A) showed lower PCR product levels in the *mdx* compared to control hearts (Fig. 1C,D). The PCR products from np 3553 to np 9990 (Fig. 1A) were also lower in the *mdx* mice (Fig. 1C,D). Levels of mitochondrial proteins including VDAC1, SDHA, Riske, and HSP60, were similar between *mdx* and control mice (Fig. 1E,F), indicating that cardiac mitochondrial content was not changed in the *mdx* mice heart. Thus, *mdx* mice at this age had much more deleted mtDNA than did control mice.

To examine whether cardiac cells with deleted mtDNAs contained high ROS levels, we stained cardiac sections with dihydroethidium (DHE), a superoxide indicator. The intensity of DHE fluorescence was significantly increased 4.6-fold in the *mdx* compared with the control mouse heart (Fig. 1G,H). The cardiac mRNA levels of the atrial (*Nppa*) and type-B (*Nppb*) natriuretic peptide genes were significantly higher in the *mdx* than in control mice (Fig. 1I), suggesting increased myocardial damage in *mdx* mice.

**Figure 2:**
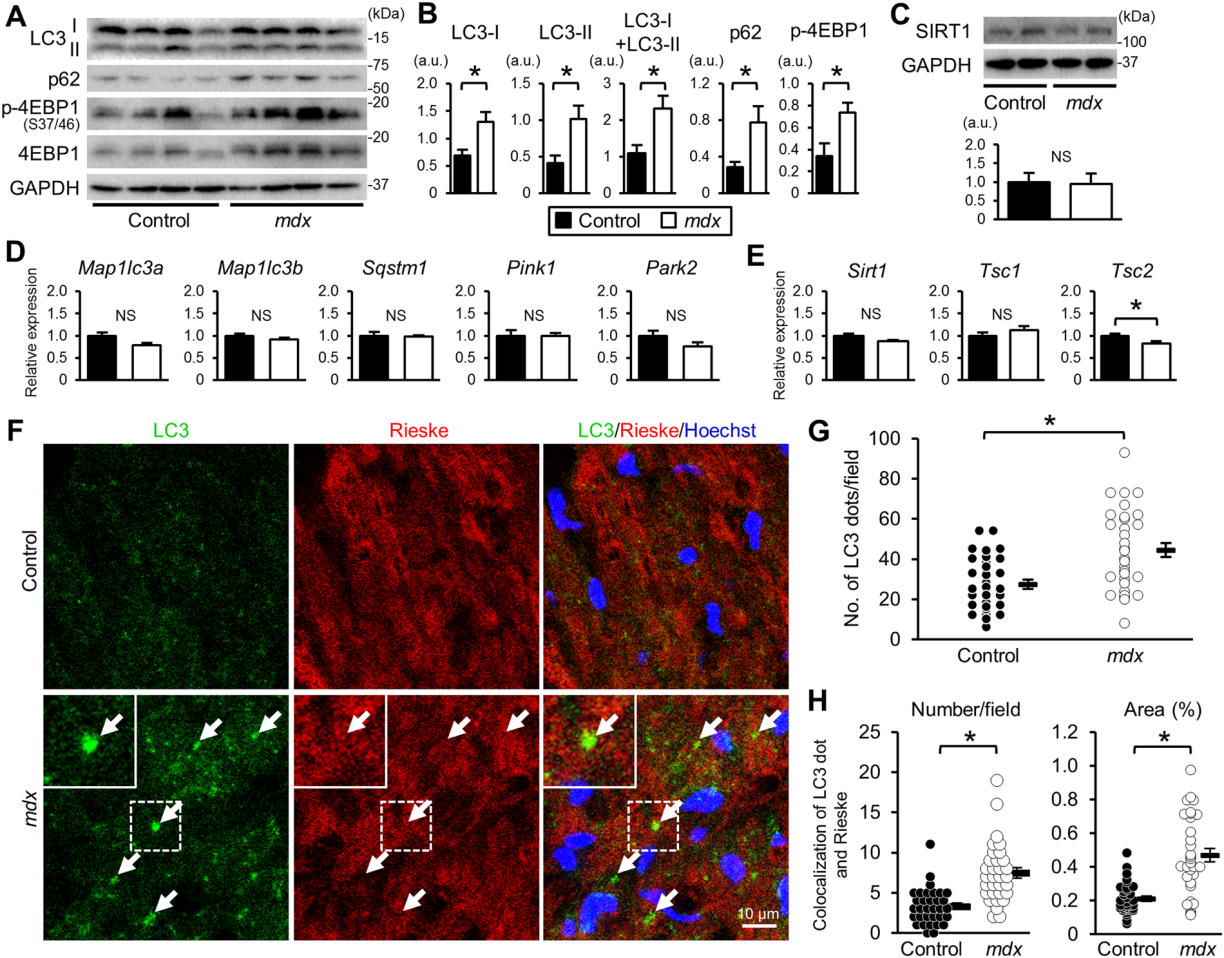
Autophagy-mediated elimination of mitochondria is disturbed in the *mdx* mouse heart. (**A**) Immunoblots for LC3, p62, phospho- (P) 4EBP1, total 4EBP1, and GAPDH in myocardium from control and *mdx* mice at 22 weeks of age. (**B**) Immunoblot data normalized to the GAPDH level. N = 4. (**C**) Representative immunoblots for SIRT1 and quantification of SIRT1 protein level normalized to GAPDH level. Expression levels of genes related to autophagy, mitophagy (**D**) and *Sirt1* and *Tsc1/2* (**E**) in myocardium. N = 4. Data in (**B**) to (**E**) were analyzed by unpaired 2-tailed Student’s t test. (**F**) Representative immunofluorescence staining for LC3 (green) and Rieske, a protein of mitochondrial complex III (red), and nuclear staining by Hoechst33342 (blue) in the control and *mdx* mouse heart. Arrows indicate LC3 dots co-localized with Rieske, i.e., autophagosomes containing mitochondria. (**G**) Number of LC3 dots per 19,600 μm^2^ in the heart. Data were analyzed by unpaired two-tailed Welch’s t-test. (**H**) Number (left) and % area (right) of co-localized LC3 dots and Rieske in heart sections analyzed using 32 randomly selected images from 4 hearts in each group. Data in number and % area were analyzed by two-tailed Welch’s t-test and Rank Sum test, respectively. *P < 0.05. NS: not significant. a.u.: arbitrary units. kDa: kilodalton.

An inhibition of autophagy has been reported in the skeletal muscles of *mdx* mice^10^. We observed that the protein levels of LC3-I, LC3-II, and p62 were increased in the *mdx* compared with the control mouse heart (Fig. 2A,B) while the mRNA levels of the *Map1lc3a*, *Map1lc3b*, and *Sqstm1* genes, which encode LC3A, LC3B, and p62, respectively, did not differ (Fig. 2D). These findings suggested that the autophagic flux was suppressed at later stages, resulting in autophagosome accumulation in the *mdx* mice. A mechanistic target of rapamycin complex 1, mTORC1, which is a negative regulator of autophagy, is reported to be activated in the skeletal muscle of *mdx* mice^10^. We found that the phosphorylation levels of eIF4E-binding protein-1 (4EBP1) (Fig. 2A,B) and S6 ribosomal protein (S6) (Supplementary Fig. 1A), downstream targets of mTORC1, were increased in the *mdx* mouse heart. On the other hand, protein and mRNA levels of SIRT1, an activator of autophagy, were not changed in the heart of *mdx* mice (Fig. 2C,E). Expression levels of mitophagy-related genes, *Pink1* and *Park2*, which encode PTEN-induced putative kinase 1 (PINK1) and parkin, respectively, were not altered in the *mdx* mice heart (Fig. 2D). *Tsc2*, a negative regulator of mTORC1, was downregulated in the *mdx* mice, though *Tsc1* expression (Fig. 2E) and phosphorylation level of Akt, an upstream activator of mTORC1, (Supplementary Fig. 1B) were unchanged. To examine mitophagy, we analyzed the co-localization of autophagosomes and mitochondria by immunostaining with anti-LC3 and anti-Rieske antibodies, which stain autophagosomes and mitochondria, respectively (Fig. 2F). The number of LC3 dots was significantly increased in the *mdx* mouse heart (Fig. 2F,G), consistent with the immunoblotting data (Fig. 2A,B). The co-localization of LC3 dots with fragmented mitochondria was significantly increased in the *mdx* compared with control mice (Fig. 2F,H). These findings indicated that the elimination of damaged mitochondria via mitophagy was disturbed, resulting in accumulation of damaged mitochondria in the *mdx* mouse heart.

### Resveratrol promotes mitophagy by facilitating autophagosome degradation

**Figure 3:**
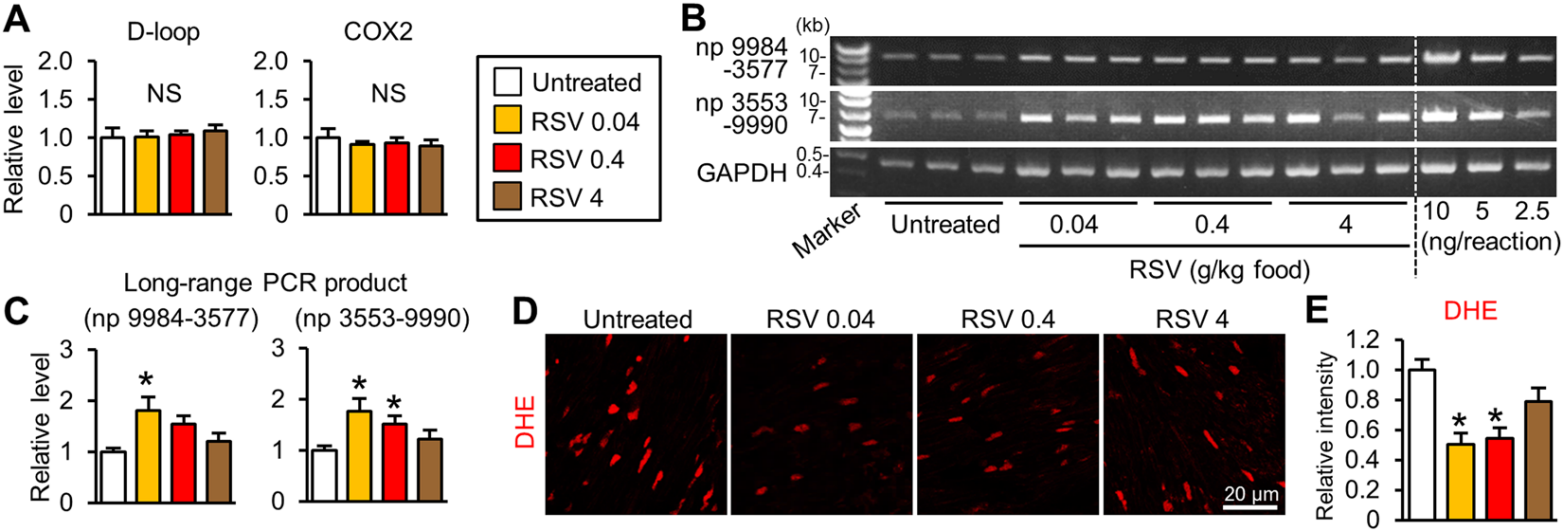
Resveratrol decreases damaged mtDNA levels in the *mdx* mice. (**A**) Quantification of mtDNA content at the D-loop and COX2 regions normalized to the RPS18 genome region in the hearts from untreated *mdx* mice and from *mdx* mice treated with resveratrol (RSV) at 0.04, 0.4, and 4 g/kg food. N = 4~5. (**B**) Representative gel images of long-range PCR of mtDNA [nucleotide positions (np) 9984-3577 and np 3553–9990]. (**C**) Levels of long-range PCR products normalized to *Gapdh*. N = 4. (**D**) Dihydroethidium (DHE) images of heart sections. (**E**) DHE fluorescence intensity per field in 12 randomly selected images from 4 hearts in each group. Data were analyzed by one-way ANOVA with a Student-Newman-Kuels method for multiple comparisons. *P < 0.05 vs. untreated *mdx* mice. NS: not significant. kb: kilobase.

To examine whether resveratrol, a SIRT1 activator, facilitates the mitophagy-mediated elimination of damaged mitochondria, we treated *mdx* mice with resveratrol (0, 0.04, 0.4, or 4 g/kg food) for 56 weeks starting at 9 weeks of age; the dose was estimated to be approximately 0, 5, 50, or 500 mg resveratrol/kg body weight/day, respectively. We previously observed that left ventricular (LV) systolic function determined by echocardiography was unchanged in *mdx* mice at 40 weeks of age compared with age-matched control mice^17^. To investigate whether resveratrol preserves LV systolic function in *mdx* mice, we investigated the effect of resveratrol at 15 months of age when LV systolic function is reported to be reduced^20^. Total 24 *mdx* mice were randomly assigned to the 4 groups (6 mice/group). During the experiments, 2, 1, 1, and 2 mice were died from muscle tumors or unknown causes in untreated, 0.04, 0.4, and 4 g resveratrol/kg food groups, respectively. There was no statistical difference in mortality rates among groups. We examined the cardiac tissues when the mice were 65 weeks old. Although resveratrol did not change the D-loop or COXII mtDNA region levels (Fig. 3A), it increased the levels of long-range PCR products in the *mdx* mouse heart (Fig. 3B,C), indicating that resveratrol promoted the elimination of abnormal mitochondria with deleted mtDNAs. Consistent with these findings, DHE staining intensities in the cardiomyocytes of *mdx* mice treated with 0.04 and 0.4 g resveratrol/kg food were about half of those of untreated *mdx* mice (Fig. 3D,E).

We previously reported that expression levels of nicotinamide adenine dinucleotide phosphate (NADPH) oxidase 4 (NOX4), which generates superoxide anion, were upregulated in the skeletal muscle from *mdx* mice^16^. *Nox4* expression level was significantly increased in the heart of *mdx* mice compared with control mice (Supplementary Fig. 2A). Resveratrol did not change *Nox4* expression levels (Supplementary Fig. 2B), suggesting that that the reduction in oxidative stress by resveratrol was independent of NOX4.

**Figure 4:**
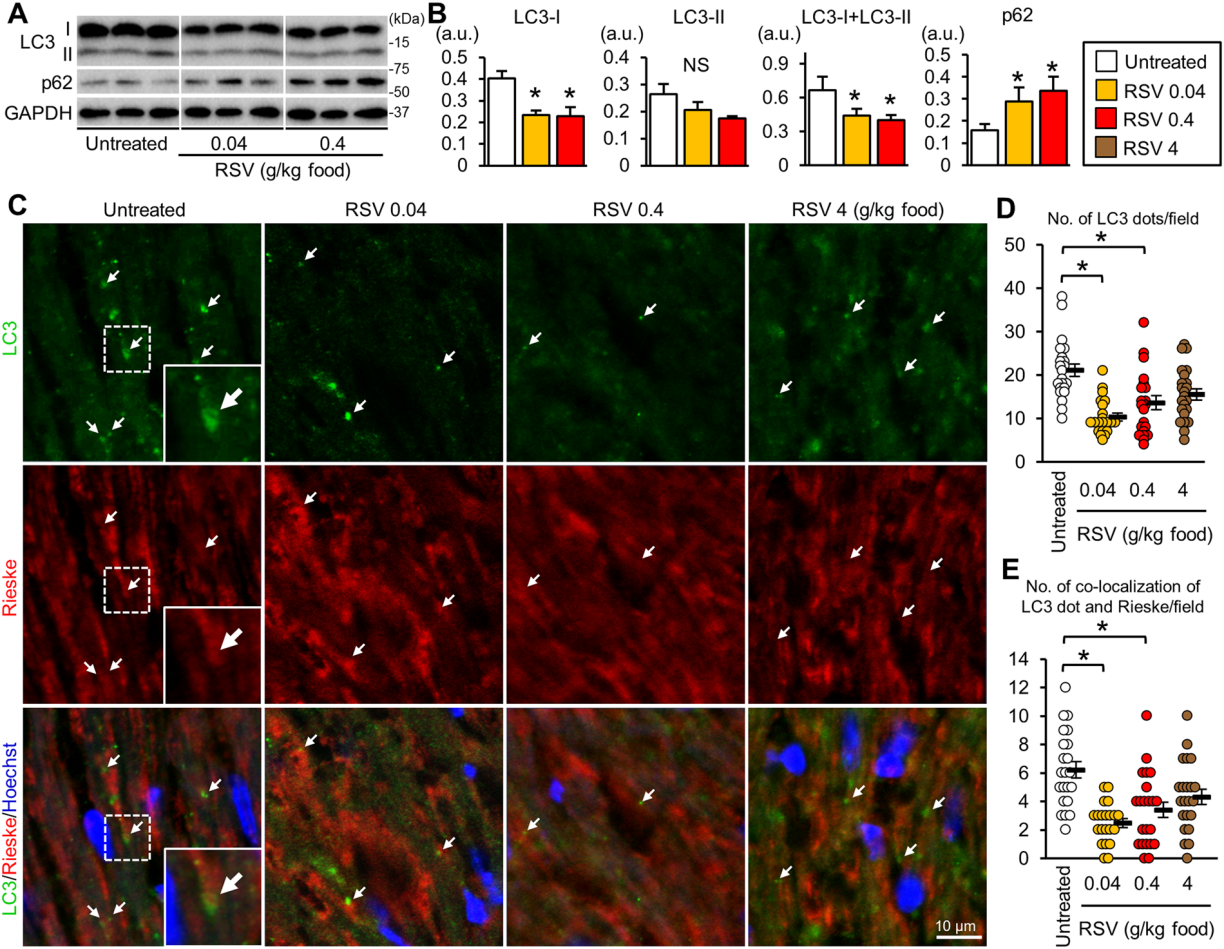
Resveratrol promotes the autophagy-mediated elimination of mitochondria in *mdx* mice. (**A**) Immunoblots for LC3 and p62 in the hearts from untreated *mdx* mice and from *mdx* mice treated with resveratrol (RSV) at 0.04, 0.4, and 4 g/kg food. Samples were run on the same gel but were not contiguous. (**B**) Protein levels. N = 4~5. *P < 0.05 vs. untreated *mdx* mice. (**C**) Immunofluorescence images for LC3 (green), Rieske (red), and merged images of LC3, Rieske, and Hoechst33342 (blue) in heart sections. Arrows indicate LC3 dots co-localized with Rieske. (**D**,**E**) Numbers of LC3 dots (**D**) and of co-localized LC3 and Rieske dots (**E**) per 4,900 μm^2^ in heart sections. We analyzed 21~22 randomly selected images from 4 hearts in each group. Data in (**B**) were analyzed by one-way ANOVA with a Student-Newman-Kuels method for multiple comparisons. Data in (**D**) and (**E**) were analyzed by one-way ANOVA on Ranks with a Dunn’s method for multiple comparisons. *P < 0.05. NS: not significant. a.u.: arbitrary units. kDa: kilodalton.

**Figure 5:**
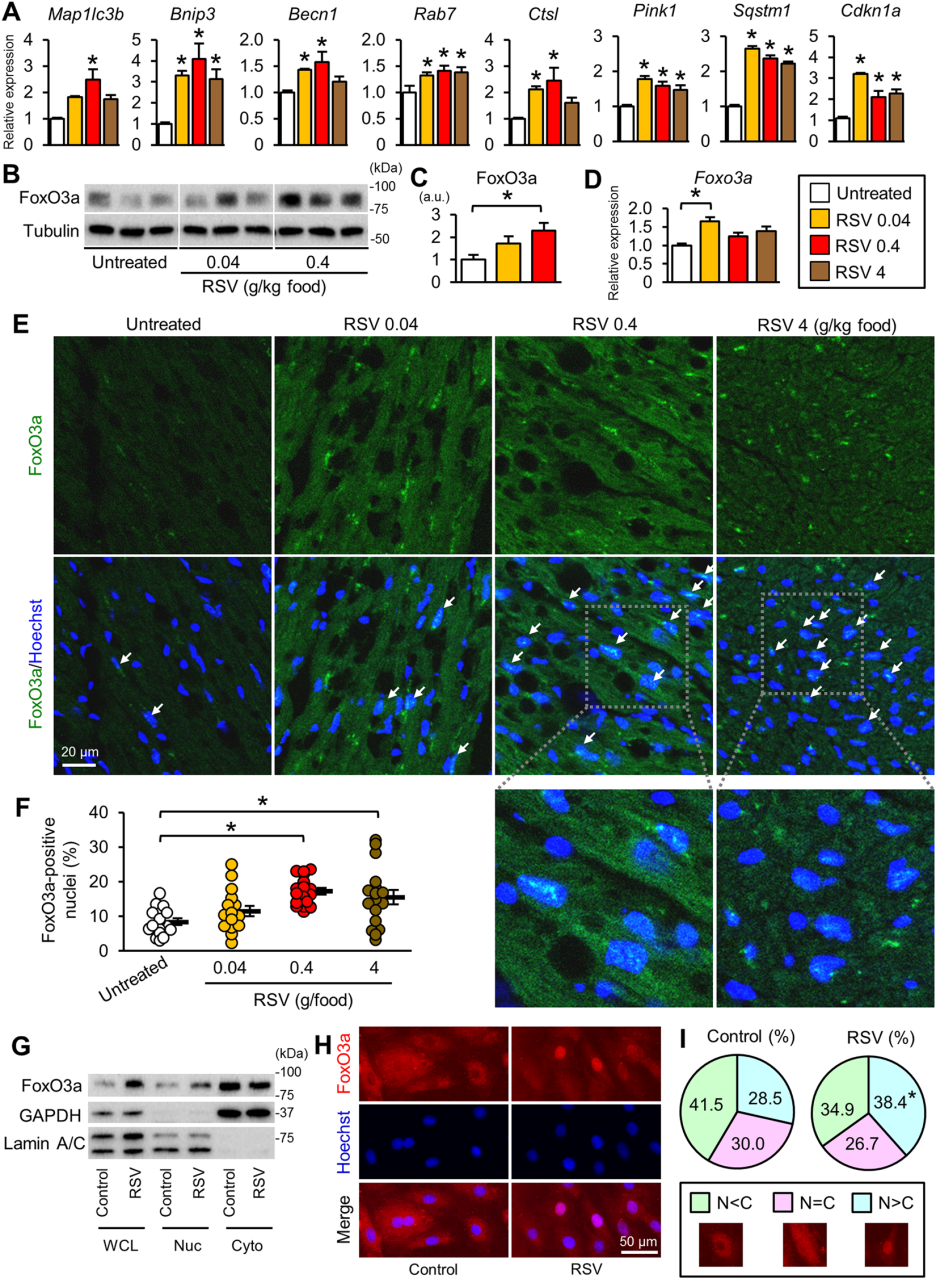
Activation of FoxO3a by resveratrol in the *mdx* mouse heart. (**A**) Expression levels of autophagy-related genes that are targets of FoxOs. N = 4~5. *P < 0.05 vs. untreated *mdx* mice. (**B**) Immunoblots for FoxO3a in the myocardium from untreated and resveratrol (RSV)-treated *mdx* mice. Samples were run on the same gel but were not contiguous. (**C**) FoxO3a protein levels. N = 4~5. (**D**) Transcript levels of the *Foxo3a* gene in the myocardium. N = 4~5. (**E**) Representative immunostaining for FoxO3a (green) and nuclear staining by Hoechst33342 (blue) in heart sections. Arrows indicate FoxO3a-positive nuclei. (**F**) Number of FoxO3a-positive nuclei. We analyzed 17 randomly captured images from three hearts in each group. *P < 0.05. (**G**) Representative immunoblots for Foxo3a, GAPDH (a cytosolic marker), and Lamin A/C (a nuclear marker) in H9c2 cells treated with vehicle (control) and resveratrol (RSV, 30 μM) for 24 hours followed by subcellular fractionation. WCL: whole cell lysate, Nuc: nuclear fraction, Cyto: cytosolic fraction. (**H**) Representative immunostaining for FoxO3a and Hoechst33342 nuclear staining in H9c2 cells treated with resveratrol as in G. (**I**) Pie charts show the proportion of FoxO3a subcellular localization. Total 200 and 232 cells were analyzed in control and resveratrol-treated cells, respectively. N < C, localized predominantly in the cytosol, N = C: localized equally in the nucleus and cytosol, N > C: localized predominantly in the nucleus. Representative images of FoxO3a staining for patterns of subcellular localization were also shown. *P < 0.05 vs. Control. Data in (**A**,**C**,**D** and **F**) were analyzed by one-way ANOVA with a Student-Newman-Kuels method for multiple comparisons. The difference in the proportions of FoxO3a localization was analyzed by the z-test. a.u.: arbitrary units. kDa: kilodalton.

We next investigated whether resveratrol restores the autophagic activity in *mdx* mice. Resveratrol at 0.04 and 0.4 g/kg food significantly reduced the protein levels of LC3-I and total LC3 (LC3-I + LC3-II) (Fig. 4A,B). Because the mRNA levels of Map1lc3B, which encodes the LC3 protein, were actually increased by resveratrol (Fig. 5A), the resveratrol-induced reduction in LC3 protein probably resulted from its degradation via autophagy in the *mdx* heart. Unexpectedly, resveratrol increased the p62 protein level (Fig. 4A,B, Supplementary Fig. 2A,B). It should be noted that a more than 2-fold increase in the mRNA level of *Sqstm1*, which encodes p62, was found in the resveratrol-treated *mdx* mice (Fig. 5A). Immunostaining showed that the numbers of LC3 dots and of LC3 dots co-localized with Rieske were significantly decreased by resveratrol at 0.04 and 0.4 g/kg food (Fig. 4C–E). The changes in total LC3 levels (Supplementary Fig. 3) and LC3 dots (Fig. 4C,D) by 4 g resveratrol/kg food did not reach statistical significance. Thus, the administration of 0.04 and 0.4 g resveratrol/kg food to *mdx* mice promoted the clearance of autophagosomes containing damaged mitochondria with deleted mtDNA.

### Resveratrol activates FoxO3a in *mdx* mice

Because FoxOs, which induce expression of autophagy/mitophagy-related genes, are positively regulated by SIRT1^21,22^, we analyzed autophagy/mitophagy-related target genes of FoxOs. We found that resveratrol significantly upregulated autophagy-related genes, including *Map1lc3B*, *Bnip3*, *Becn1*^13^, *Rab7*^23^, *Pink1*^13^, *Sqstm1*^24^, and *Ctsl*^25^, which are transactivated by FoxOs (Fig. 5A). *Cdkn1a*, a FoxO target that is not involved in autophagy, was also upregulated by resveratrol (Fig. 5A). Because FoxO3a has been reported to play a role in cardiac autophagy^14,15^, we further analyzed whether FoxO3a was activated by resveratrol treatment. The protein levels of FoxO3a were increased by resveratrol, especially at the dose of 0.4 g/kg food (Fig. 5B,C). The mRNA levels of *Foxo3a* were also upregulated by resveratrol (Fig. 5D). The nuclear localization of FoxOs is required for their transcriptional activity, and SIRT1 activation or resveratrol is reported to induce the nuclear retention of FoxO1^26^ and FoxO3a^27^. Immunostaining revealed that the number of FoxO3a-positive nuclei in cardiomyocytes was significantly higher in the *mdx* mice treated with resveratrol at 0.4 and 4 g/kg food than in untreated *mdx* mice (Fig. 5E,F). To further confirm whether resveratrol treatment induces nuclear localization of FoxO3a, we utilized H9c2 cardiomyocytes. Treatment of cells with resveratrol increased the FoxO3a protein level in the whole cell lysate as well as in the nuclear fraction and decreased it in the cytosolic fraction (Fig. 5G). In addition, immunocytochemistry showed that resveratrol treatment significantly increased FoxO3a localized in the nucleus (Fig. 5H,I). These results suggested that resveratrol increased the activity of FoxO3a.

### Resveratrol increases SIRT1 activity without changing mTORC1 activity

**Figure 6:**
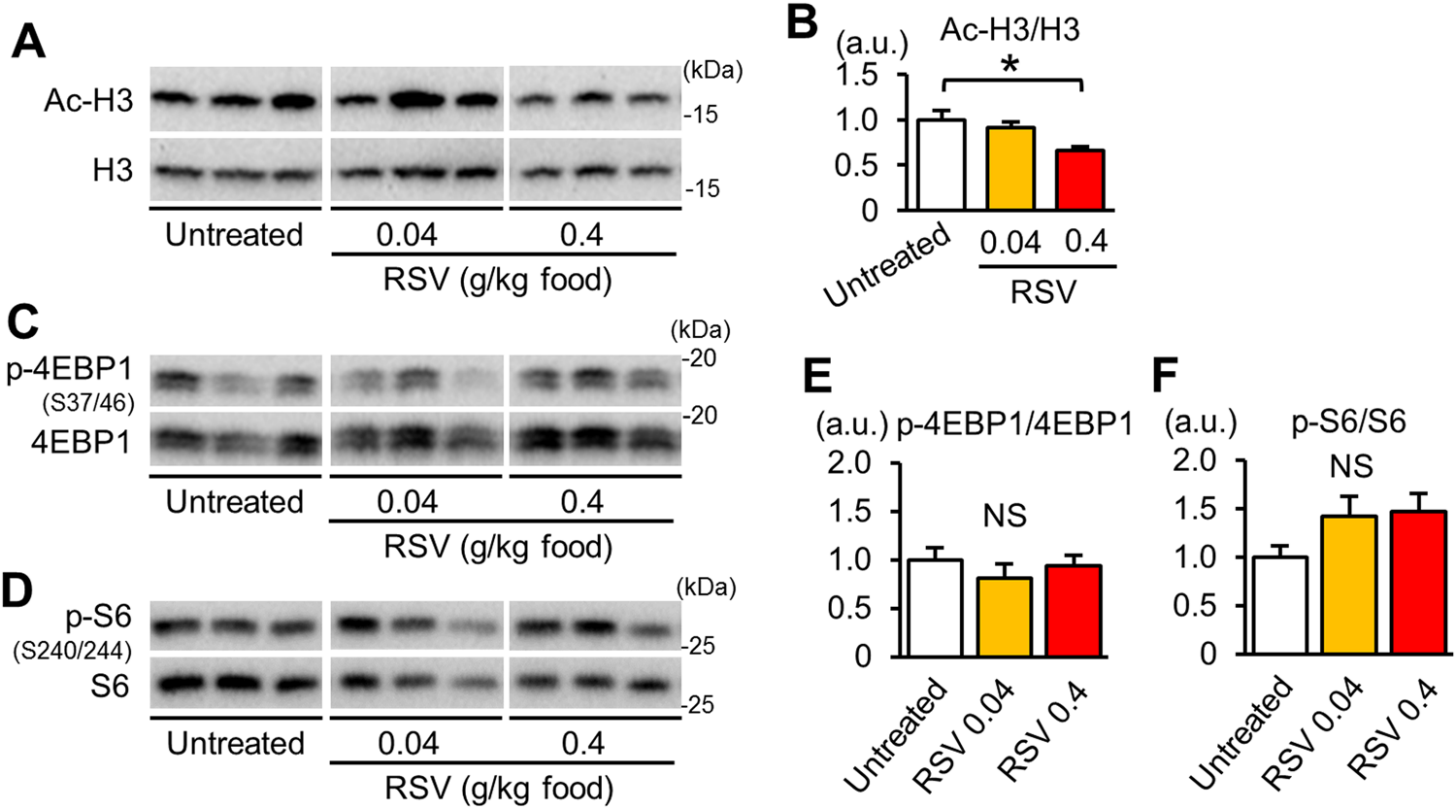
Effects of resveratrol on SIRT1 and mTORC1 activity. (**A**) Representative immunoblots for acetyl-histone H3 (Ac-H3) and total histone H3 in the myocardium. Samples were run on the same gel but were not contiguous. (**B**) Protein levels of acetyl-histone H3 normalized to total histone H3. (**C**,**D**) Representative immunoblots for phospho (p)-Ser37/46-4EBP1 (**C**) and p-Ser240/244-S6 (**D**) in myocardium from untreated and resveratrol (RSV)-treated *mdx* mice. Samples were run on the same gel but were not contiguous. Summary data of levels of p-4EBP1 normalize to total 4EBP1 (**E**) and p-S6 normalized to total S6 levels (**F**). All data were analyzed by one-way ANOVA with a Student-Newman-Kuels method for multiple comparisons. *P < 0.05. NS: not significant. a.u.: arbitrary units. kDa: kilodalton.

We investigated whether resveratrol treatment increases SIRT1 activity in the heart of *mdx* mice. Acetylation levels of histone H3, one of deacetylation targets of SIRT1, were decreased in the heart of *mdx* mice treated with 0.4 g resveratrol/kg food compared with that of untreated *mdx* mice (Fig. 6A,B). Although mRNA levels of SIRT1 were significantly increased by 0.04 and 0.4 g resveratrol/kg food (Supplementary Fig. 4A), resveratrol treatment did not change SIRT1 protein levels (Supplementary Fig. 4B–E). These results suggested that catalytic activation of SIRT1 by resveratrol was important for its function in the heart of *mdx* mice. Although it has been reported that SIRT1 and resveratrol suppress mTOR activity^28,29^, levels of phospho-4EBP1 and phospho-S6 were not affected by either dose of resveratrol (Fig. 6C–F, supplementary Fig. 3C–F).

### Resveratrol ameliorates the cardiomyopathy in *mdx* mice

**Figure 7:**
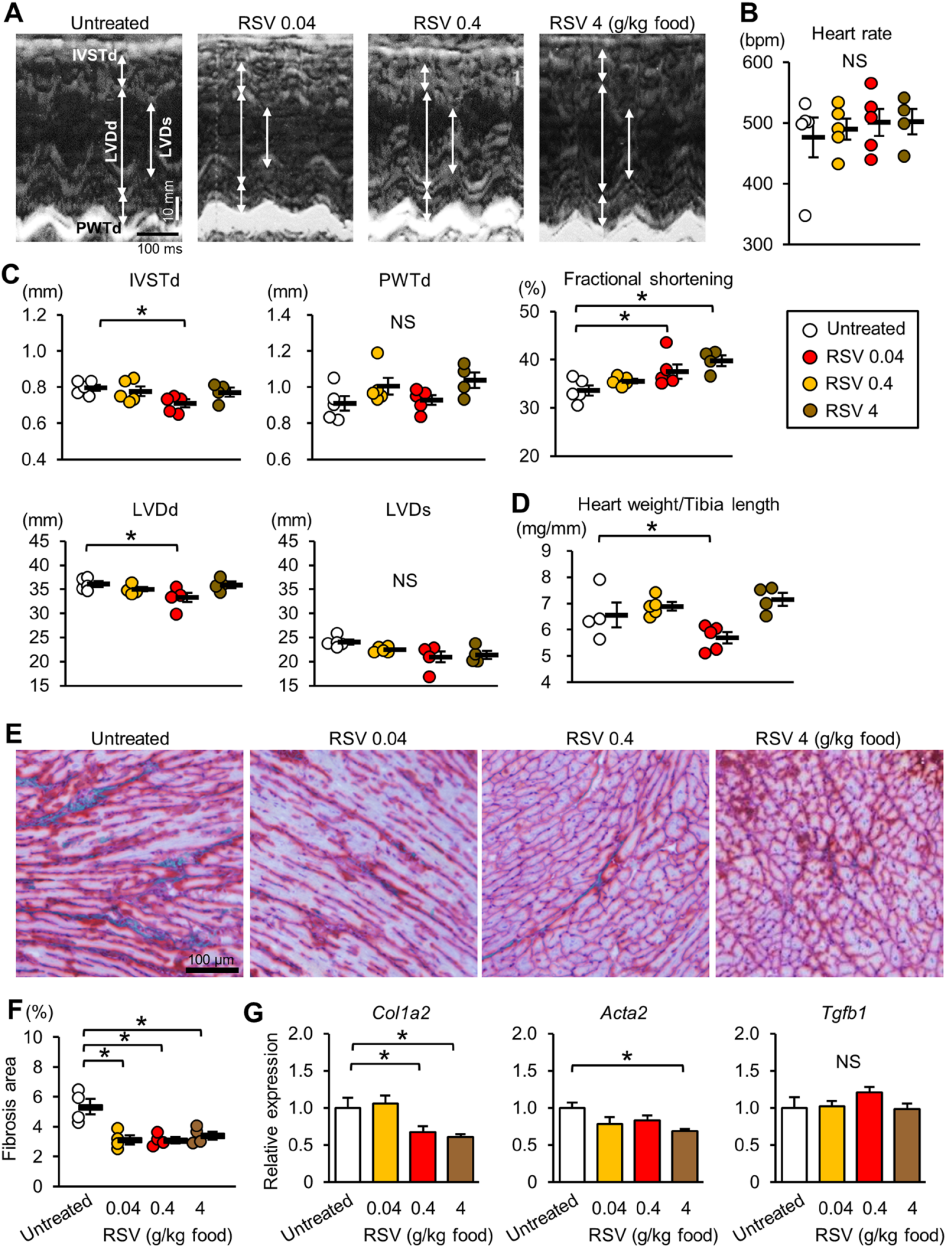
Resveratrol ameliorates the cardiomyopathy in *mdx* mice. (**A**) Representative echocardiograms (M-mode view) of untreated and resveratrol (RSV)-treated 62-week-old *mdx* mice. Heart rate during echocardiography (**B**) and echocardiographic measurements (**C**). N = 4~5. IVSTd: interventricular septum thickness at diastole, PWTd: left ventricular posterior wall thickness at diastole, LVDd: left ventricular end-diastolic diameter, LVDs: left ventricular end-systolic diameter. (**D**) Heart weight to tibia length ratio of untreated and RSV-treated 65-week-old *mdx* mice. N = 4~5. (**E**) Representative Masson Trichrome staining in the heart sections of *mdx* mice. (**F**) Summary data of the percentage of fibrosis area. Data were analyzed from 4 hearts in each group. (**G**) Expression of *Col1a2* (encoding the pro-alpha2 chain of type I collagen), *Acta2* (encoding α-smooth muscle actin), and *Tgfb1* (encoding transforming growth factor β1). Data except for (**F**) were analyzed by one-way ANOVA with a Student-Newman-Kuels method for multiple comparisons. Data in (**F**) were analyzed one-way ANOVA on Ranks with a Dunn’s method for multiple comparisons. N = 4~5. *P < 0.05. NS: not significant.

*Mdx* mice have been reported to show LV dilation and systolic dysfunction at 12 months of age^20^. During echocardiography of mice at 62-week-old (Fig. 7A), heart rate was comparable among 4 groups (Fig. 7B). Echocardiography (Fig. 7C) revealed that the interventricular septal thickness (IVST) was thinner and the end-diastolic LV dimensions (LVDd) were smaller in mice treated with 0.4 g resveratrol/kg food than in untreated *mdx* mice. Fractional shortening, an index of LV systolic function, was significantly increased by resveratrol at 0.4 and 4 g/kg food. Consistent with the echocardiography data, the heart weight to tibia length ratio at 65 weeks old was smaller in mice treated with 0.4 g resveratrol/kg food (Fig. 7D). Resveratrol did not affect the LV posterior wall thickness (PWT).

Masson Trichrome staining showed that fibrosis area was significantly decreased by resveratrol at any doses (Fig. 7E,F). The mRNA levels of *Col1a2*, which encodes collagen Iα2, were decreased by resveratrol at 0.4 and 4 g/kg food (Fig. 7D). The mRNA levels of *Acta2*, which encodes α-smooth muscle actin (αSMA), a marker of myofibroblasts that produce the extracellular matrix, were decreased by resveratrol treatment, and especially the reduction by resveratrol at 4 g/kg food reached statistical significance (Fig. 7D). On the other hand, resveratrol did not change mRNA levels of *Tgfb1*, which encodes transforming growth factor β-1 (TGFβ1), in the heart of *mdx* mice.

## Discussion

We found that the *mdx* mouse heart at 22 weeks of age had lower levels of intact mtDNA by the long-range PCR method (Fig. 1C,D), indicating accumulation of abnormal mitochondria with deleted mtDNAs in the *mdx* mouse heart. The increased levels of total LC3 and p62 proteins (Fig. 2A,B) and autophagosomes containing mitochondria (Fig. 2F,H) suggest that disturbed autophagic elimination of mitochondria underlies accumulation of damaged mitochondria in the *mdx* heart. The accumulation of abnormal mitochondria could explain ROS production observed in the *mdx* mice (Fig. 1G,H) and also cause metabolic imbalances, leading to cardiac insufficiency.

Reductions in long-range PCR products of mtDNA observed in *mdx* mice (Fig. 1C,D) were not due to a decrease in the mitochondrial content, because mtDNA contents measured by the qPCR method and levels of mitochondria proteins evaluated by immunoblotting were preserved in the *mdx* heart (Fig. 1B,E and F). In contrast to the previous report^19^, our data suggest that mtDNA deletions seemed not to be site-specific because long-range PCR products of two different regions (np 9984-3577 and np 3553–9990, Fig. 1A) were equally decreased in the *mdx* heart (Fig. 1C,D). In addition, both D-loop and COXII regions of mtDNA were slightly, but not significantly, decreased in *mdx* mice (Fig. 1B), supporting the notion that deletion occurred at random, but not site-specific. Because resveratrol treatment similarly increased the long-range PCR products of both two regions (Fig. 3B,C), the reductions in mtDNA deletion by resveratrol was also site-nonspecific. The significance of these site-nonspecific mtDNA deletions in mitochondrial function remains unclear and warrants further investigation.

We found that LC3-II level and the number of LC3 dots examined by immunoblotting and immunostaining, respectively, were increased in the *mdx* heart (Fig. 2A,B,F and G). The increase in autophagosomes can be interpreted as either increased autophagosome formation or suppressed degradation of autophagosomes^8^. In this study, another autophagy marker p62 protein level was also increased in the *mdx* mice (Fig. 2A,B). In addition, mRNA levels of LC3 and p62 were unchanged in *mdx* mice (Fig. 2D), indicating that elevations in LC3 and p62 protein levels were not attributed to transcriptional changes. Collectively, these findings suggest that autophagic activity was suppressed at the processes of autophagosome-lysosome fusion and/or lysosomal autophagosome degradation.

mTORC1 inhibits autophagosome-lysosome fusion^30^ and the lysosomal degradation of autophagosomes^31^. In addition, the hyperactivation of mTORC1 by *Tsc2* knockout in cardiomyocytes results in accumulations of both autophagosomes and abnormal mitochondria^32^. Therefore, activated mTORC1 (Fig. 2A,B, Supplementary Fig. 1A) may have contributed to the suppression of autophagosome degradation in the *mdx* mice. Resveratrol promoted autophagy and mitophagy in the *mdx* mice (Fig. 4) without changing the mTORC1 activity (Fig. 6C–F). Instead, resveratrol induced the expression of FoxO3a and the nuclear localization of FoxO3a (Fig. 5B–I) and upregulated the mRNAs of autophagy-related genes that are targets of FoxOs (Fig. 5A), suggesting that resveratrol activated autophagy and mitophagy via FoxO activation. Among the genes upregulated by resveratrol, *Rab7* and *Ctsl* may contribute to the restoration of autophagic flux, because Rab7 participates in autophagosome-lysosome fusion^33^, and cathepsin L is a lysosomal hydrolase.

Our data support the notion that suppressed autophagy caused enhancement of tissue oxidative stress via impaired elimination of damaged mitochondria. It has been reported that NOX4 which generates superoxide anion is upregulated in the heart^34^ and skeletal muscle of *mdx* mice^16^ and therefore could be a source of superoxide anion in tissues. In the present study, *Nox4* expression was expectedly upregulated in the heart of *mdx* mice compared with that of control mice (Supplemental Fig. 2A). However, *Nox4* expression levels were not changed by resveratrol treatment (Supplemental Fig. 2B). Therefore, it seems that resveratrol reduced the oxidative stress independent of NOX4 although we cannot exclude the involvement of upregulated NOX4 in oxidative stress in the *mdx* heart. Nevertheless, resveratrol could reduce oxidative stress and improved pathophysiological conditions of the heart, suggesting that the major source of ROS in the *mdx* mouse heart was damaged mitochondria.

The most effective dose was expected to be 0.4 g resveratrol/kg food. However, a dose as low as 0.04 g resveratrol/kg food was effective for mitophagy induction and decreased cardiac ROS levels, which were comparable to the results obtained with 0.4 g/kg food (Figs 3E and 4E). We note that resveratrol is hydrophobic, and may accumulate in lipids. Thus, the long-term administration of resveratrol may have led to the efficacy of the 0.04 g/kg food dose. On the other hand, the maximum benefit was not obtained by the highest dose of resveratrol. It is reported that resveratrol acts as a mitochondrial depolarizing agent^35^. As described above, resveratrol is hydrophobic and therefore may be excessively accumulated in tissues especially at the highest dose. In addition, our data showed that the reduction in ROS was partial in the mice treated with the highest dose of resveratrol (Fig. 3D,E). Therefore, we cannot exclude the possibility that the highest dose of resveratrol might partly cancel beneficial effects via such an unfavorable action.

An anti-fibrotic function of resveratrol was found at any doses in *mdx* mice (Fig. 7E,F). Resveratrol treatment did not change expression levels of TGFβ, a powerful inducer of fibrosis (Fig. 7G). This was consistent with our previous report showing that resveratrol attenuated fibrosis in the skeletal muscle of *mdx* mice without changing TGFβ expression^16^. In addition, αSMA expression levels were reduced by resveratrol, suggesting that resveratrol attenuated myofibroblast differentiation, which is stimulated by TGFβ. Therefore, it seems that resveratrol targets signaling downstream of the TGFβ receptor to suppress fibrosis. Since it is recently reported that FoxO1 plays a role in the shift of the TGFβ’s action from profibrotic toward anti-inflammatory^36^, modulation of TGFβ signaling by FoxOs may underlie the reduction in fibrosis afforded by resveratrol.

The primary cause of the myopathy in DMD is a fragility of cellular membranes^1^. We propose here that a disturbance in mitophagy induces an acquired mitochondriopathy that further produces cellular ROS and contributes to cardiomyopathy development in DMD. Therefore, mitophagy-promoting therapies are effective for DMD. A low-protein diet and mTORC1 inhibitors improve the muscle phenotype of dystrophic mice^9,10^. Since mTORC1 is a master regulator of translation, its long-term inhibition would disturb muscular protein synthesis, which could exacerbate the pathophysiology of dystrophies. Because resveratrol did not inhibit mTORC1 (Fig. 6), resveratrol and other SIRT1 activators have the advantage of activating autophagy without affecting mTORC1 activity in treating dystrophic cardiomyopathy.

## Methods

### Animals and resveratrol treatment

Mice were handled according to Guide for the Care and Use of Laboratory Animals, published by the US National Institutes of Health (NIH publication No. 85-23, revised 1996) and this study was approved by the Animal Use Committee of Sapporo Medical University. Male *mdx* mice and age-matched control C57BL10 mice were purchased from Oriental Yeast Co. Ltd. (Tokyo, Japan). In a first series of experiments, we analyzed cardiac tissues in *mdx* and control C57BL10 mice at 22 weeks old. In another series of experiments, *mdx* mice were orally treated with resveratrol (Food grade, CharmaDex, Irvine, CA, USA) ad libitum mixed with powdered food (0.04, 0.4, or 4 g/kg food) starting at 9 weeks of age. The dose was estimated to be approximately 0, 5, 50, or 500 mg resveratrol/kg body weight/day, respectively. Mice left untreated served as untreated control. The mice were sacrificed at the age of 65 weeks, and cardiac tissues were sampled and examined.

### Echocardiography

At 62 weeks of age, echocardiography was performed under anesthesia with isoflurane, using Vivid-i ultrasound (GE Healthcare, Waukesha, WI, USA) with an 11.5-MHz probe. The LV was assessed in the parasternal long-axis view by an echocardiographer blind to animal groups. The interventricular septal thickness, LV posterior wall thickness, and LV dimension were measured from M-mode tracings of LV obtained at the mid-papillary muscle level with a sweep speed of 50 mm/sec.

### Analysis of gene expression by real-time quantitative PCR

Total RNA was isolated from cardiac tissues by using RNeasy Fibrous Tissue Mini Kit (Qiagen, Valencia, CA, USA). Two micrograms of total RNA per sample were reverse transcribed into cDNA, and real-time quantitative PCR were performed by using GoTaq® 2-step RT-qPCR System (Promega, Madison, WI, USA) in StepOne real-time PCR system (Thermo Fisher Scientific, Waltham, MA, USA). Each sample was run in duplicate, and the mean value was used to calculate the mRNA levels of the gene of interest and the housekeeping reference gene (Actb or Gapdh encoding β-actin or GAPDH, respectively). For each sample, the mRNA levels of the gene of interest were normalized to β-actin or GAPDH using the standard curve method. The primer sequences are listed in Supplementary Table 1.

### Immunoblotting

Immunoblotting was performed as previously reported^16,17^. The antibodies used were mouse monoclonal anti-VDAC1 (ab14734), mouse monoclonal anti-SDHA (ab14715), mouse monoclonal anti-Rieske (ab14746), mouse monoclonal anti-SIRT1 (ab110304), rabbit polyclonal anti-histone H3 (ab1791) from Abcam (Cambridge, UK); mouse monoclonal anti-HSP60 (SPA-806) from Enzo Life Sciences (Farmingdale, NY); guinea pig polyclonal anti-p62 (GP62-C) from Progen (Heidelberg, Germany); rabbit monoclonal anti-LC3A/B (#12741), rabbit monoclonal anti-phospho-Ser37/46-4EBP1 (#2855), rabbit polyclonal anti-4EBP1 (#9452), rabbit polyclonal anti-phospho-ser235/236 S6 (#2211), rabbit monoclonal anti-S6 (#2217), rabbit polyclonal anti-phospho-Ser473 Akt (#9271), rabbit polyclonal anti-Akt (#9272), rabbit monoclonal anti-FoxO3a (#2497), Lamin A/C (#2032) from Cell Signaling Technologies (Danvers, MA, USA); mouse monoclonal anti-GAPDH (G8795), mouse monoclonal anti-α-tubulin (T5168) from Sigma Aldrich (St. Louis, MO, USA); and rabbit polyclonal anti-acetylated histone H3 (382158) from Millipore (Billerica, MA, USA).

### Histological analysis

Frozen heart sections were prepared for immunostaining as previously described^17^. To detect autophagosomes and mitochondria by Immunostaining, we used rabbit anti-LC3A/B (Cell Signaling Technologies, #12741) and mouse anti-Rieske (Abcam, ab14746), respectively. Tissue sections were fixed with 4% paraformaldehyde, blocked, and incubated with rabbit anti-LC3A/B (1:200) and mouse anti-Rieske (1:100) overnight at 4 °C. LC3A/B and Rieske were detected by AlexaFluor488 anti-rabbit and AlexaFluor594 anti-mouse antibodies (Thermo Fisher Scientific), respectively. The rabbit antibody against FoxO3a (Cell Signaling Technologies, #2497, 1:250) and Hoechst33342 (Dojindo, Kumamoto, Japan, 346–07951) was used to detect nuclear localization of FoxO3a protein in the *mdx* mice heart.

To analyze the tissue superoxide level as a marker of oxidative stress, heart slices were stained with DHE (Sigma Aldrich, D1168) which is converted to ethidium bromide by superoxide and generates red fluorescence in the nucleus. Frozen tissue sections were incubated with 5 µM DHE in PBS at 37 °C for 30 min followed by nuclear staining by Hoechst33342. Fluorescence of DHE and Hoechst33342 was imaged by the confocal microscopy (Carl Zeiss, Jena, Germany) under the same conditions. Fluorescence intensity was analyzed by using ImageJ.

Cardiac fibrosis was examined by Masson Trichrome staining. Images were taken on a BZ-X700 microscope (Keyence). The percentage of fibrosis area was determined by using ImageJ as previously reported^37^. At least 8 randomly captured images were analyzed and averaged in each heart.

### Analyses of mtDNA content and deletion

Genomic DNA was isolated from mouse ventricular myocardium by using QIAamp DNA Mini kit (Qiagen, 51304). mtDNA content was quantified by a qPCR method relative to nuclear DNA copy number. To amplify mtDNA and nuclear DNA, StepOne real-time PCR system (Thermo Fisher Scientific) and GoTaq qPCR Master Mix (Promega, A6001) were used. The primer sequences at mtDNA COX2 and D-loop regions and nuclear genome RPS18 region are listed in Supplementary Table 1. To analyze levels of deletion with mtDNA, long-range PCR^38^ was performed using KOD FX Neo (Toyobo, Osaka, Japan, KFX-201). Primer sequences for long-range PCR were listed in Supplementary Table 1. In each reaction, 5 ng of DNA sample was applied for long-range PCR in 50 μl of reaction volume. The PCR was performed under the following condition: one cycle of 2 min at 94 °C, followed by 18 cycles of 98 °C for 10 sec; 65 °C for 30 sec, and 72 °C for 10 min. To verify whether this PCR condition is reliable for quantification, 10, 5, and 2.5 ng of DNA samples from an intact mouse heart were amplified together with unknown samples. Agarose gel electrophoresis was used to detect PCR products. As an internal control, nuclear GAPDH gene was also amplified.

### Experiments using cultured H9c2 cells

H9c2 cardiomyocytes were cultured with Dulbecco’s modified Eagle’s medium supplemented with 1% antibiotic-antimycotic mixed stock solution and 10% fetal bovine serum. Cells were treated with vehicle or resveratrol (30 μM) for 24 hours. Nuclear/Cytosol Fractionation Kit (K266-100, BioVision) was used to extract nuclear and cytosolic fractions. In immunoblotting, Lamin A/C and GAPDH were used as markers of the nuclear and the cytosol fractions, respectively. For FoxO3a immunocytochemistry, cells were fixed with 4% paraformaldehyde followed by incubation of cells for 30 minutes with PBS containing 3% bovine serum albumin, 1% goat serum, and 0.3% Triton X-100. Cells were then incubated with antibodies against FoxO3a (1:250) overnight at 4 °C, washed with PBS, incubated with anti-rabbit IgG conjugated with AF594 antibodies (1:2000 dilution) for 1 hours at room temperature, and washed again with PBS. Samples were then incubated with Hoechst33342 (1:1000 dilution) to stain the nucleus. Fluorescence images were obtained using a FLoid Cell Imaging Station (Thermo Fisher Scientific). The patterns of intracellular localization of FoxO3a were divided into three; predominantly localized in the nucleus, localized equally in the nucleus and the cytosol, and predominantly localized in the cytosol.

### Statistics

Data are expressed as means ± SEM. An unpaired 2-tailed Student’s *t*-test or Mann-Whitney Rank Sum test was used to compare two groups. To compare multiple groups, we used one-way ANOVA followed by a post-hoc Student-Neuman-Keuls test or Kruskal-Wallis one-way ANOVA on Ranks with Dunn’s method. Mortality of resveratrol-treated *mdx* mice was analyzed by chi-square test. The *z*-test was used to determine the significance in proportions of subcellular localization of FoxO3a between two groups. Differences were considered significant if the P-value was less than 0.05. All analyses were performed with SigmaStat (Systat Software, San Jose, CA, USA).

The datasets generated during the current study are available from the corresponding author on reasonable request.

## Electronic supplementary material

Supplementary Figures and Table
