# Supplementary material for: Resveratrol Ameliorates Mitophagy Disturbance and Improves Cardiac Pathophysiology of Dystrophin-deficient mdx Mice
Source: Sci Rep. 2018 Oct 22;8:15555. doi: 10.1038/s41598-018-33930-w (PMC6197260; doi:10.1038/s41598-018-33930-w)
Supplement: Supplementary file 1 — Supplementary Figures and Table [file 41598_2018_33930_MOESM1_ESM.pdf]

Supplementary Files

Resveratrol Ameliorates Mitophagy Disturbance and Improves Cardiac Pathophysiology of Dystrophin-deficient *mdx* Mice

Atsushi Kuno, Ryusuke Hosoda, Rio Sebori, Takashi Hayashi, Hiromi Sakuragi, Mika Tanabe, Yoshiyuki Horio

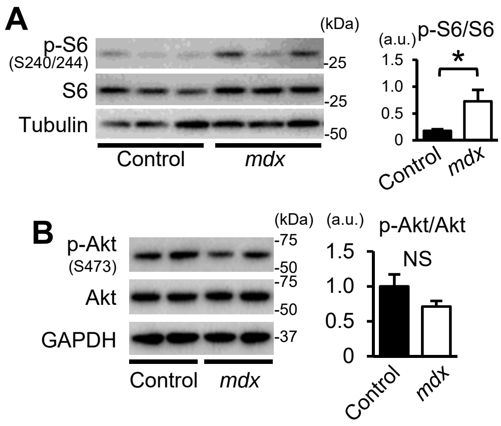

Supplementary Figure 1. mTORC1 activity in the *mdx* mice heart.

(A) Immunoblots (left) and summary data (right) for phospho (P)-S6 at Ser240/244 and total S6 in myocardium from control and *mdx* mice at 22 weeks of age. N=4. (B) Immunoblots (left) and summary data (right) for phospho (P)-Ser473-Akt and total Akt in the heart. Data were analyzed by unpaired 2-tailed Student's t test. N=4. *P<0.05. NS: not significant. a.u.: arbitrary units.

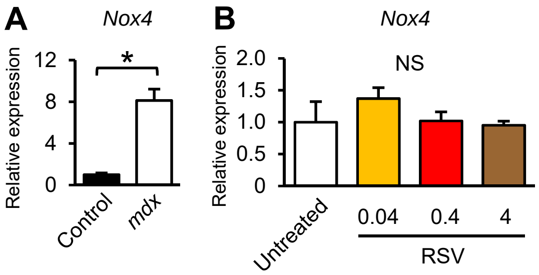

Supplementary Figure 2. NOX4 expression in the *mdx* mice heart.
(A) NOX4 mRNA levels in myocardium from control and *mdx* mice at 22 weeks of age. Data were analyzed by unpaired 2-tailed Student's t test. N=4. (B) NOX4 mRNA levels in myocardium from untreated *mdx* mice and *mdx* mice treated with resveratrol (RSV) at 0.04, 0.4, and 4 g/kg food. Data were analyzed by one-way ANOVA. N=4-5 in each group. *P<0.05. NS: not significant.

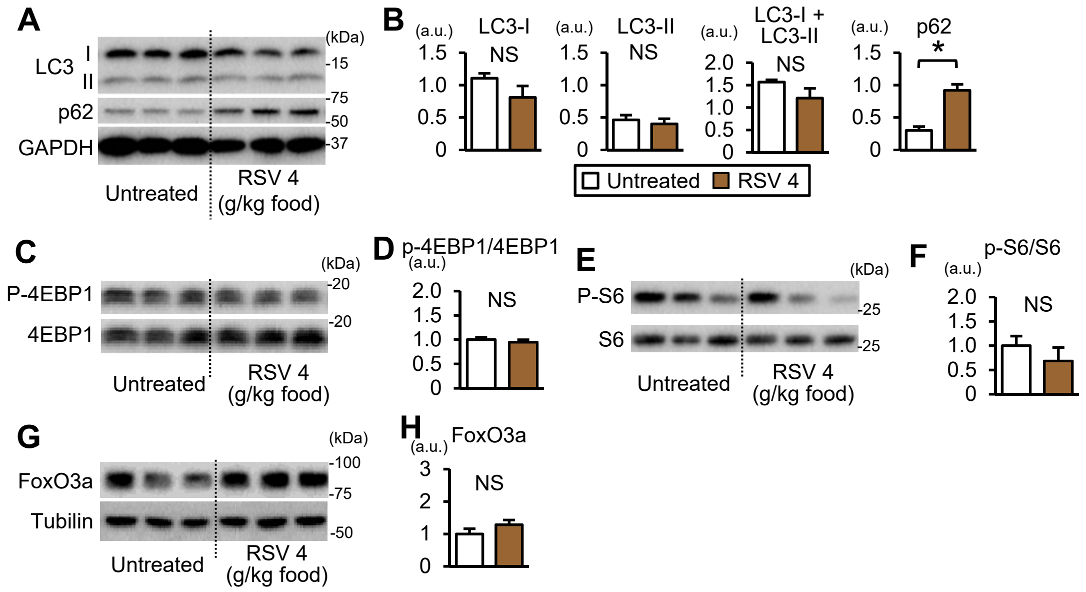

Supplementary Figure 3. Effects of 4 g resveratrol/kg food on autophagic activity, mTORC1 activity, FoxO3a, and SIRT1 expression in the *mdx* mice heart.

(A) Representative immunoblots for LC3 and p62 in the hearts of untreated *mdx* mice and *mdx* mice treated with resveratrol at 4 g/kg food. (B) Summary of protein levels of LC3 and p62. N=4. Representative immunoblots for phospho (P)-4EBP1 (C) and P-S6 (E). Summary data of levels of P-4EBP1 (D) and P-S6 (F) normalized to total proteins were shown. N=4. (G) Representative immunoblots for FoxO3a. N=4 in each group. (H) Summary of FoxO3a protein levels. *P<0.05. NS: not significant. a.u.: arbitrary units. . Data were analyzed by unpaired 2-tailed Student's t test.

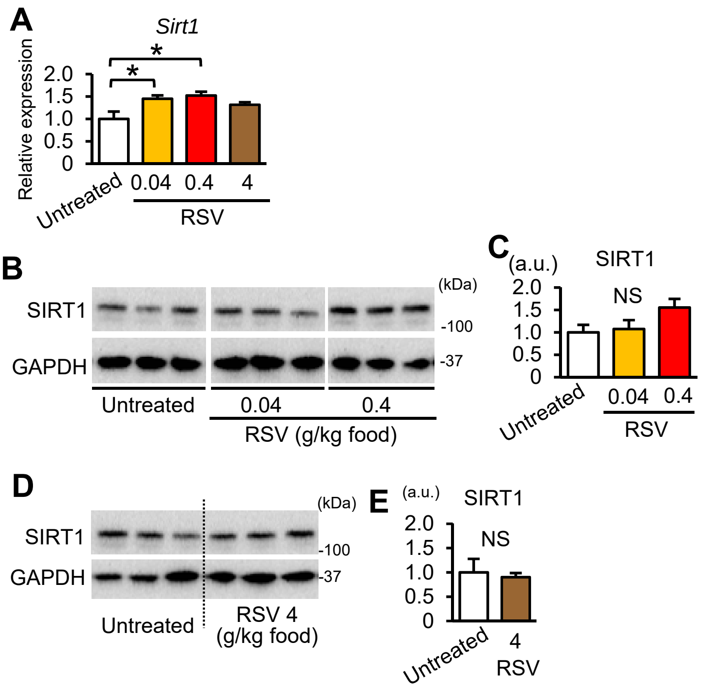

Supplementary Figure 4. Effects of resveratrol on SIRT1 expression in the *mdx* mice heart.
(A) SIRT1 mRNA levels determined by a qPCR method. (B and D) Representative immunoblots for SIRT1 in myocardium from untreated *mdx* mice and *mdx* mice treated with resveratrol (RSV) at 0.04, 0.4, and 4 g/kg food. (C and E) Quantification of SIRT1 protein levels normalized to GAPDH levels. Data in (A) and (B) were analyzed by one-way ANOVA. Data in (E) were analyzed by unpaired 2-tailed Student's t test. N=4-5 in each group. *P<0.05. NS: not significant. a.u.: arbitrary units.

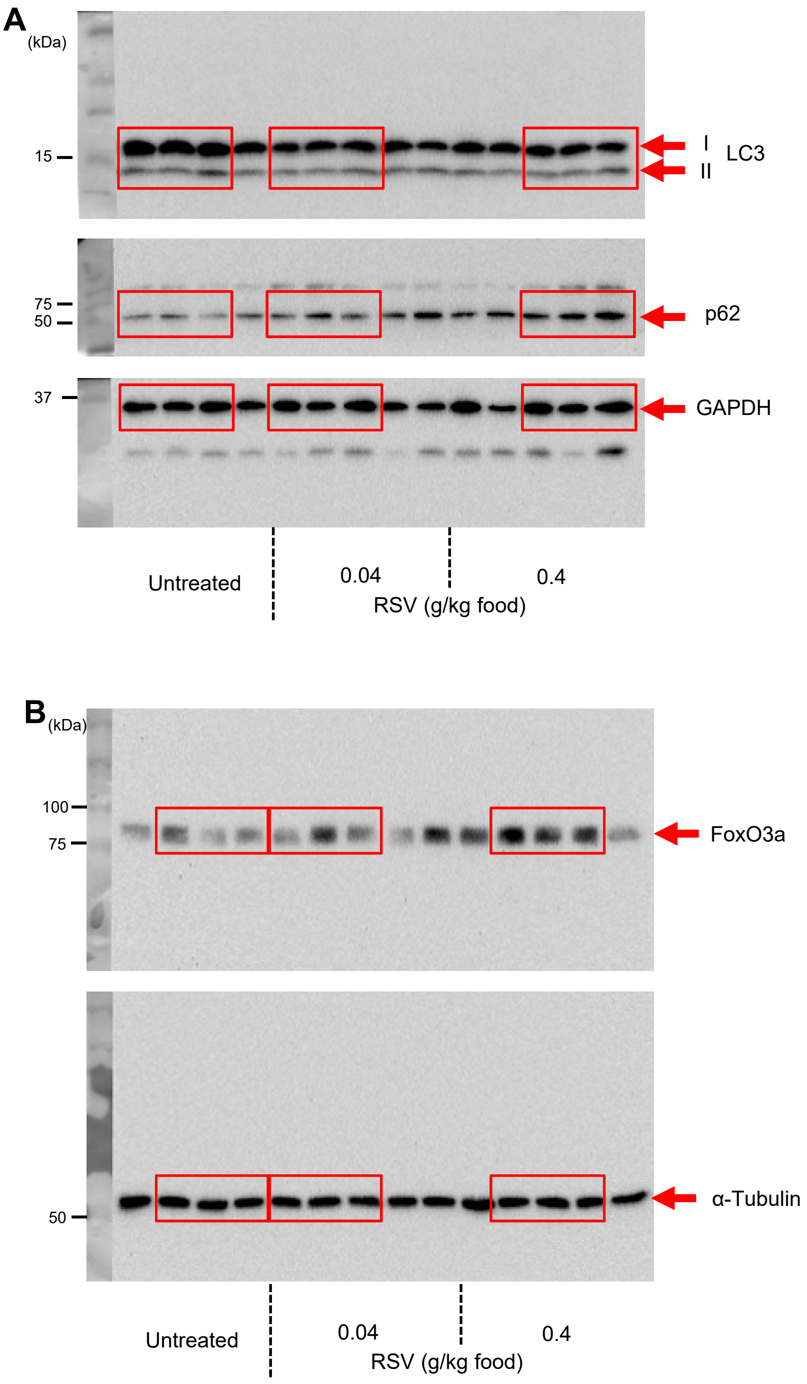

Supplementary Figure 5. Unprocessed western blots for Figure 4A (A), and Figure 5B (B).

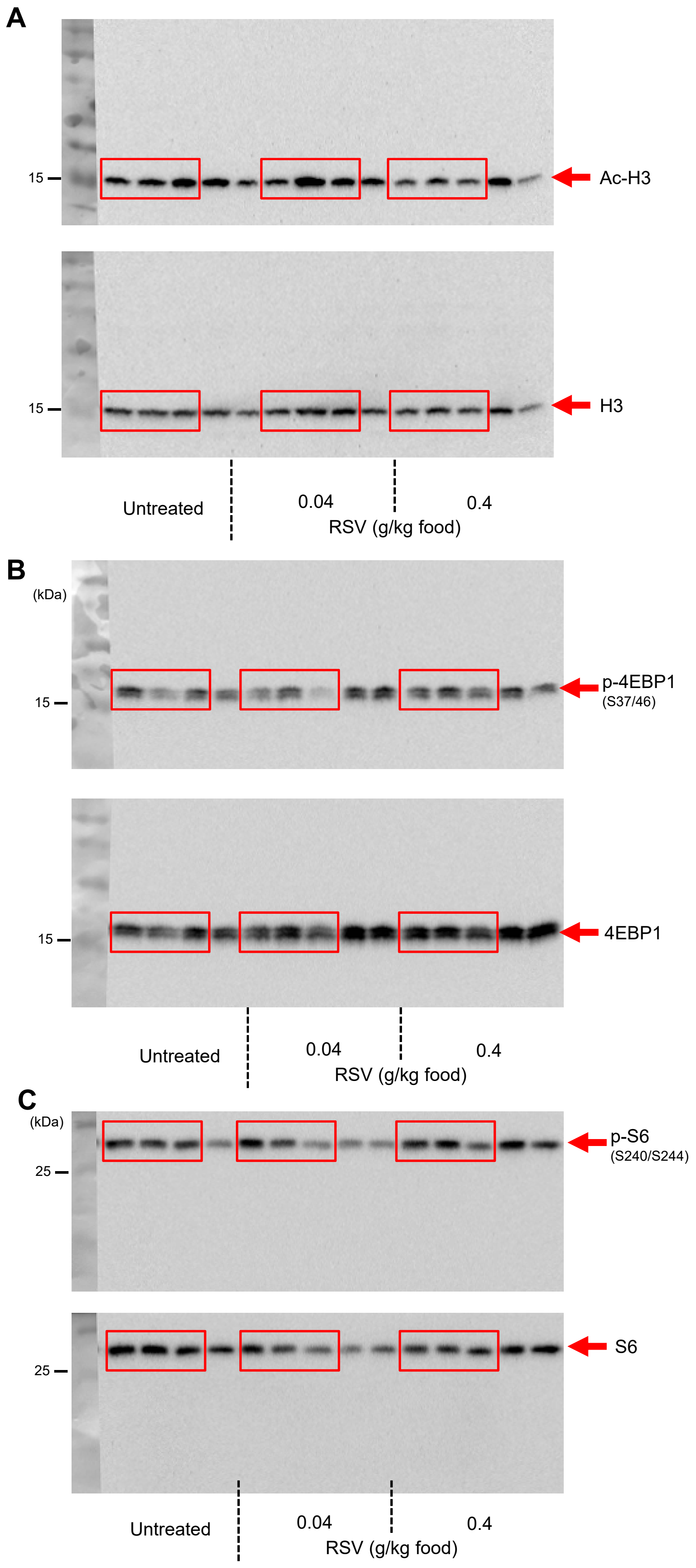

Supplementary Figure 6. Unprocessed western blots for Figure 6A (A) F igure 6C (B), and Figure 6D (C).

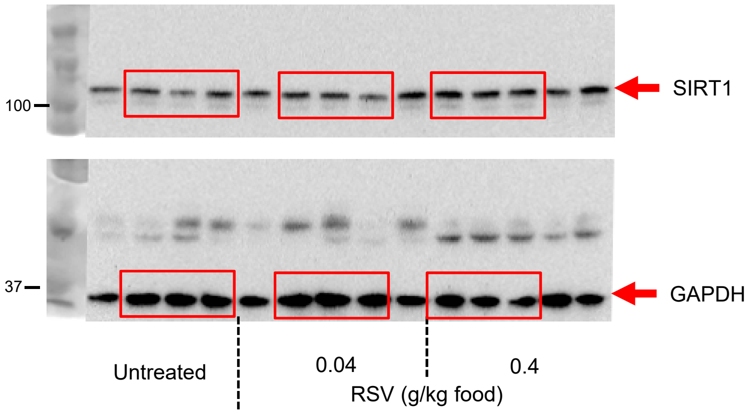

Supplementary Figure 7. Unprocessed western blots for Supplementary Figure 4B.

Supplementary Table 1. Primer sequences for PCR analyses

Gene	Forward primer (5’ to 3’)	Reverse primer (5’ to 3’)
For expression assay		
Map1lc3a	CTATGAACAGGAGAAGGATGAAG	ACTCAGAAGCCGAAGGTT
Map1lc3b	CGTCCTGGACAAGACCAAGT	ATTGCTGTCCCGAATGTCTC
Sqstm1	CCTTGCCCTACAGCTGAGTC	CACACTCTCCCCCACATTCT
Col1a2	TGTTGGCCCATCTGGTAAAGA	CAGGGAATCCGATGTTGCC
Acta2	ACTCTCTTCCAGCCATCTTTCA	ATAGGTGGTTTGGTGGATGC
Tgfb1	TGGAAAGGGCCCAGCAC	GCAATAGTTGGTATCCAGGGCT
Bnip3	TCCACTAGCACCTTCTGATGA	GAACACCGCATTTACAGAACAA
Becn1	CTGACAGACAAATCTAAGGAG	AATAGGAGCCGCCACTGCCTC
Rab7	TGAACCCATCAAACTGGACA	GAGGAGGGACGCATATTGAA
Ctsl	TGACACAGGGTTCGTGGATA	CTTGCGTCCATAGCAACAGA
Pink1	TGAGGAGCAGACTCCCAGTT	AGTCCCACTCCACAAGGATG
Parkin	TGGAAAGCTCCGAGTTCAGT	CCTTGTCTGAGGTTGGGTGT
Cdkn1a	TCCACAGCGATATCCAGACA	GGACATCACCAGGATTGGAC
Foxo3a	AACAGACCAGCCACCTTCTCTT	GCTGACAGAATTTGACAAGGCA
Nppa	ATTGACAGGATTGGAGCCCAGAGT	TGACACACCACAAGGGCTTAGGAT
Nppb	CTGAAGGTGCTGTCCCAGAT	GTTCTTTTGTGAGGCCTTGG
Sirt1	GACGCTGTGGCAGATTGTTA	GGAATCCCACAGGAGACAGA
Tsc1	CCTGACACCACCAAGGAAGT	TGGAGAAGGTGGCTTCTGTT
Tsc2	CCTGGGCAATGACTTTGTTT	TCCATGTCTTTCCTGCACTG
Gapdh	TCACCACCATGGAGAAGGC	GCTAAGCAGTTGGTGGTGCA
Actb	CTGGCTCCTAGCACCATGAAGAT	GGTGGACAGTGAGGCCAGGAT
For mtDNA analyses		
mtDNA (COX2)	ATAACCGAGTCGTTCTGCCAAT	TTTCAGAGCATTGGCCATAGAA
mtDNA (D-loop)	GCGTTATCGCCTCATACGTT	GATTGGGTTTTGCGGACTAA
nDNA (RPS18)	TGTGTTAGGGGACTGGTGGACA	CATCACCCACTTACCCCCAAAA
Long-range PCR (np 9984-3577)	CCATCTACCTTCTTCAACCTCACC	GATGCTCGGATCCATAGGAATGTTG
Long-range PCR (np 3553-9990)	CAACATTCCTATGGATCCGAGCATC	GGTGAGGTTGAAGAAGGTAGATGG
GAPDH	ACCACAGTCCATGCCATCAC	TCCACCACCCTGTTGCTGTA
